# Rocaglates as Antivirals: Comparing the Effects on Viral Resistance, Anti-Coronaviral Activity, RNA-Clamping on eIF4A and Immune Cell Toxicity

**DOI:** 10.3390/v14030519

**Published:** 2022-03-03

**Authors:** Wiebke Obermann, Alexandra Friedrich, Ramakanth Madhugiri, Paul Klemm, Jan Philipp Mengel, Torsten Hain, Stephan Pleschka, Hans-Guido Wendel, Roland K. Hartmann, Susanne Schiffmann, John Ziebuhr, Christin Müller, Arnold Grünweller

**Affiliations:** 1 Institute of Pharmaceutical Chemistry, Philipps University Marburg, 35032 Marburg, Germany; wiebkeobermann@staff.uni-marburg.de (W.O.); klemmp@staff.uni-marburg.de (P.K.); roland.hartmann@staff.uni-marburg.de (R.K.H.); 2 Institute of Medical Virology, Justus Liebig University Giessen, 35392 Giessen, Germany; alexandra.friedrich@viro.med.uni-giessen.de (A.F.); ramakanth.madhugiri@viro.med.uni-giessen.de (R.M.); stephan.pleschka@viro.med.uni-giessen.de (S.P.); john.ziebuhr@viro.med.uni-giessen.de (J.Z.); 3 Institute of Medical Microbiology, Justus Liebig University Giessen, 35392 Giessen, Germany; jan.p.mengel@mikrobio.med.uni-giessen.de (J.P.M.); torsten.hain@mikrobio.med.uni-giessen.de (T.H.); 4 German Center for Infection Research, Partner Site Giessen-Marburg-Langen, 35392 Giessen, Germany; 5 Cancer Biology and Genetics Program, Memorial Sloan Kettering Cancer Center, New York, NY 10023, USA; wendelh@mskcc.org; 6 Fraunhofer Institute for Translational Medicine and Pharmacology, 60596 Frankfurt am Main, Germany; susanne.schiffmann@itmp.fraunhofer.de

**Keywords:** rocaglates, coronavirus, eIF4A, silvestrol, CR-1-31-B, zotatifin, escape mutations, broad-spectrum antivirals

## Abstract

Rocaglates are potent broad-spectrum antiviral compounds with a promising safety profile. They inhibit viral protein synthesis for different RNA viruses by clamping the 5′-UTRs of mRNAs onto the surface of the RNA helicase eIF4A. Apart from the natural rocaglate silvestrol, synthetic rocaglates like zotatifin or CR-1-31-B have been developed. Here, we compared the effects of rocaglates on viral 5′-UTR-mediated reporter gene expression and binding to an eIF4A-polypurine complex. Furthermore, we analyzed the cytotoxicity of rocaglates on several human immune cells and compared their antiviral activities in coronavirus-infected cells. Finally, the potential for developing viral resistance was evaluated by passaging human coronavirus 229E (HCoV-229E) in the presence of increasing concentrations of rocaglates in MRC-5 cells. Importantly, no decrease in rocaglate-sensitivity was observed, suggesting that virus escape mutants are unlikely to emerge if the host factor eIF4A is targeted. In summary, all three rocaglates are promising antivirals with differences in cytotoxicity against human immune cells, RNA-clamping efficiency, and antiviral activity. In detail, zotatifin showed reduced RNA-clamping efficiency and antiviral activity compared to silvestrol and CR-1-31-B, but was less cytotoxic for immune cells. Our results underline the potential of rocaglates as broad-spectrum antivirals with no indications for the emergence of escape mutations in HCoV-229E.

## 1. Introduction

RNA viruses, like coronaviruses and influenza viruses, are important human pathogens and have significant zoonotic potential, facilitating the transmission of newly emerging viruses from animal reservoirs to humans. As a result, they represent a formidable challenge for global disease control, which is highlighted by the ongoing SARS-CoV-2 pandemic [1]. RNA viruses have a high genetic variability and use a plethora of mechanisms to increase their fitness, including short replication times, high yields and fast mutation rates [2]. The rapid accumulation of beneficial mutations facilitates adaptation to new environmental conditions, including the evolution of resistant variants that escape from, e.g., antiviral treatments [3]. Specifically, influenza A viruses (IAVs) were reported to acquire drug-resistance against adamantanes (e.g., rimantadine, targeting the M2 ion channel) or neuraminidase (NA) inhibitors (e.g., oseltamivir). Consequently, these inhibitors have become partially obsolete as a treatment option for IAV due to the appearance of drug-resistance mutations in M2 (and NA) [4].

Compared to IAV and other RNA viruses, the mutation rate during coronavirus genome replication is slightly lower because these viruses employ in their replication-transcription complex a 3′-to-5′ exoribonuclease that acts in conjunction with the viral RNA-dependent RNA polymerase to remove misincorporated nucleotides (proof-reading activity) [5,6]. Despite their increased replication fidelity, coronaviruses retained their capability of readily acquiring mutations under specific selection pressures, often resulting in changes in replication efficiency, disease severity, transmissibility and antigenicity. For example, genetic variants (of concern) with altered phenotypes continue to emerge in the current SARS-CoV-2 pandemic, preferentially with mutations in the spike protein gene that improve replication efficiency, transmissibility or lead to immune escape [7,8].

Thus far, very few anti-coronaviral drugs are available [9], highlighting the urgent need to develop broad-spectrum antiviral drugs against this group of RNA viruses [10]. Historically, drug development efforts mainly focused on direct-acting antivirals (DAAs) targeting viral proteins. DAAs have, in general, the advantage of shorter treatment times and fewer side effects, but can give rise to rapidly emerging drug-resistant variants, as highlighted above for IAVs. However, viruses as obligate intracellular parasites exploit numerous host proteins for their replication. There is increasing evidence that these essential proteins can be targeted in therapeutic approaches using a second class of antivirals called host-directed antivirals (HDAs). HDAs are potentially active against many (if not all) members of a given virus family or virus genus and, thus, may potentially be used as broad-spectrum antivirals [11,12]. However, HDAs may also increase the risk of side effects and less favorable safety profiles owing to interference with essential cellular processes.

A host function essential for viral replication is the protein synthesis carried out by the cellular translation machinery. In this context, the cellular DEAD-box RNA helicase eIF4A, which unwinds RNA secondary structures in 5′-untranslated regions (5′-UTRs) as part of the translation initiation complex eIF4F, has been identified as an essential factor for viral protein synthesis (reviewed in [13]). Since a large number of RNA viruses harbor highly structured 5′-UTRs, they require the unwinding activity of eIF4A to allow binding of the 43S-preinitiation complex (43S-PIC) during translation initiation [14]. Therefore, it is not surprising that pharmacological inhibition of eIF4A has been shown to efficiently prevent replication of a large set of RNA viruses, including corona-, picorna-, flavi-, filo-, hepe-, toga-, arena-, nairo-, orthomyxo- and bunyaviruses [15,16,17,18,19,20,21,22,23].

A very promising class of eIF4A inhibitors (and therefore HDAs) are rocaglates, a group of flavaglines that can clamp the 5′-UTRs of viral and selected cellular mRNAs onto the eIF4A surface. This RNA-clamping prevents the unwinding of mRNA secondary structures by eIF4A and, consequently, translation initiation [24]. Since the translation of many proto-oncogenes is eIF4A-dependent, some of these selective rocaglates are in pre-clinical and early-stage clinical cancer studies [25,26,27,28]. One prominent example is zotatifin, a synthetic rocaglate that successfully reached clinical trials aimed to establish therapeutics against advanced solid tumor malignancies (phases 1–2), and also entered an efficacy and dose-escalating study in patients with mild or moderate COVID-19 (phase 1b) [25], which laid the foundations for the further development of rocaglates as potential pan-antivirals.

In the study presented here, the potential to develop rocaglate-resistant mutations was evaluated by multiple passages of human coronavirus 229E (HCoV-229E) in cells grown in a culture medium containing increasing concentrations of two different rocaglates. In our experimental setup, we found no reduction in the sensitivity of passaged HCoV-229E to rocaglate treatment, providing evidence that the emergence of coronaviral escape mutants is unlikely when targeting the host factor eIF4A. Moreover, we compared the effects of zotatifin as well as silvestrol and CR-1-31-B on (i) viral 5′-UTR-mediated reporter gene expression in HepG2 cells, (ii) binding to a purified human eIF4A-polypurine complex, (iii) cytotoxicity in a diverse set of primary human immune cells, and (iv) on antiviral activity in MRC-5 and in primary human bronchial epithelial cells as a relevant ex vivo cell model system.

In summary, all three rocaglates are suitable potent antiviral compounds that differ in their cell type-specific cytotoxicity, RNA-clamping efficiency and antiviral activity. In detail, zotatifin showed slightly reduced RNA-clamping and antiviral activity compared to silvestrol and CR-1-31-B but was less cytotoxic for immune cells and normal bronchial epithelial cells. Our results underline the potential of rocaglates as broad-spectrum antiviral compounds, especially for the treatment of coronaviruses, but also reveal differences between rocaglate variants regarding their antiviral activity and cytotoxicity in cells of the human immune system.

## 2. Materials and Methods

### 2.1. Cell Culture and Viruses

Human fetal lung fibroblasts (MRC-5; ATCC CCL-171), Huh-7 (Japanese Collection of Research Bioresources (JCRB) cell bank, Osaka, Japan [29]) and Vero E6 (ATCC CRL-1586) cells were grown in Dulbecco’s modified Eagle’s medium (DMEM, Invitrogen, Carlsbad, CA, USA) supplemented with 10% fetal calf serum (FCS), 100 U/mL of penicillin and 100 μg/mL of streptomycin. HepG2 cells were cultured in Iscove’s Modified Dulbecco’s Medium (IMDM) supplemented with 10% FCS. Primary human monocytes, macrophages, T cells and dendritic cells were cultured in RPMI1640 GlutaMAX medium supplemented with 10% FCS, 100 U/mL of penicillin and 100 μg/mL of streptomycin. All cells were cultured at 37 °C in a 5% CO_2_ atmosphere.

Genome sequences of coronavirus strains used in this study are as follows: HCoV-229E (NCBI accession number AF304460.1, NCBI reference sequence NC_002645.1), MERS-CoV (NCBI accession number JX869059, NCBI reference sequence NC_019843.3). The SARS-CoV-2 isolate Munich 929 [30] was kindly provided by Christian Drosten (Institute of Virology, Charité-Universitätsmedizin, Berlin, Germany).

### 2.2. Human Airway Epithelial Cells

Cryopreserved normal human bronchial epithelial (NHBE; CC-2540, donor 1: TAN 24717, Lot No. 000312626; donor 2: TAN 36585, Batch: 18TL269120) cells were obtained from Lonza, and undifferentiated cells were seeded on collagen-coated transwell plates (Corning Costar, Corning, NY, USA). Cells were grown in a mixture of DMEM and bronchial epithelial cell growth medium (BEGM, Lonza, Basel, Switzerland) supplemented with retinoic acid (75 nM, Sigma Aldrich, St. Louis, MO, USA) at 37 °C in a 5% CO_2_ atmosphere. Fresh medium was added regularly after 2 days. After reaching confluence, the cells were cultivated under air-liquid interface conditions for 4 additional weeks for full differentiation into pseudostratified human airway epithelia. Medium from the basolateral compartment was replaced with fresh medium every 2–3 days, and the apical surface was washed every week with PBS (Invitrogen, Carlsbad, CA, USA).

### 2.3. Reagents

Silvestrol was obtained from the Sarawak Biodiversity Centre (Kuching; North-Borneo, Malaysia; purity > 99%). A 6 mM stock solution was prepared in DMSO (sterile-filtered; Roth). Zotatifin (MedChemExpress, Monmounth Junction, NJ, USA; purity: 98%), CR-1-31-B (also known as CR-31-B (−)) and the inactive enantiomer CR-1-30-B (also known as CR-31-B (+), for further details see [20,31,32,33]) were dissolved in DMSO at a concentration of 10 mM. All stock solutions were stored at −20 °C and diluted in the corresponding growth media (DMEM or IMDM).

### 2.4. Cell Toxicity

Cell growth and viability of MRC-5 cells in the presence of the respective compounds were determined by the 3-(4,5-dimethylthiazol-2-yl)-2,5-diphenyl-2H-tetrazoliumbromide (MTT) method, as described previously [19]. For analyzing cytotoxicity in NHBE cells after 72 h treatment, the trans-epithelial electrical resistance (TEER) was measured using an epithelial Volt/Ohm meter 3 (EVOM3, WPI, Sarasota, FL, USA). The obtained TEER values were then compared to those obtained from untreated cells.

The OranguTM assay (Cell Guidance Systems Ltd., Cambridge, UK) was used to determine the cell viability of human monocytes, M1 and M2 macrophages, monocyte-derived dendritic cells (MdDCs) and T cells. CD14+ and CD4+ cells were isolated using the human CD14 or CD4 microbeads (Miltenyi Biotec, Bergisch Gladbach, Germany) as recommended by the supplier. CD14+ monocytes were differentiated to monocyte-derived dendritic cells, M1 macrophages or M2 macrophages, as described previously [34]. Then, 1 × 10^5^ cells were seeded in 96-well plates. Different concentrations of silvestrol, zotatifin, CR-1-31-B and CR-1-30-B (0.5–100 nM) or vehicle (DMSO) were added, and cells were incubated at 37 °C and 5% CO_2_. After 20 h (T cells) or 24 h (monocytes, MdDCs, M1 and M2 macrophages) of incubation, 10 µL of Orangu^TM^ cell counting solution was added, followed by incubation for 4 h (T cells) or 1 h (monocytes, MdDCs, M1 and M2 macrophages) at 37 °C and 5% CO_2_. After incubation, the absorbance was measured at a wavelength of 450 nm with a reference at 650 nm using an EnSpire^®^ 2300 Multimode Plate Reader (Perkin Elmer, Waltham, MA, USA). To calculate cell viability in the Orangu^TM^ assay, the absorbance of vehicle-treated cells was set to 100%, and the rocaglate-treated samples were normalized to this value correlated to them.

### 2.5. Serial Passaging of Virus

Serial passaging of virus-containing cell culture supernatants until passage (p) 15 was performed in the presence of increasing concentrations of silvestrol and CR-1-31-B (5–10 nM). As a control, the virus was passaged in the presence of the inactive enantiomer CR-1-30-B or in the absence of any inhibitor. More precisely, MRC-5 cells were infected in triplicate with HCoV-229E at a defined multiplicity of infection (MOI) of 0.1/0.01 and incubated at 33 °C for 48/72 h post-infection (hpi) in the presence of silvestrol, CR-1-31-B, CR-1-30-B or solvent control. Viral titers of the collected supernatants were determined by plaque assay on Huh-7 cells (as described in [19]). At p15, each virus stock and the original stock from p0 were used to obtain individual isolates by two rounds of end-point dilution assay. Virus stocks prepared for each of these isolates were subsequently used to infect Huh-7 cells. At 24 h hpi, total RNA was isolated from infected cells using the RNeasyKit (Qiagen, Venlo, Netherlands). Libraries for sequencing were prepared using NEB Ultra II RNA directional reagents according to the manufacturer’s instructions. Library fragments for each sample were then molecular barcoded and amplified with unique dual index primers (NEB E6440) to avoid barcode noise in sequencing. Finally, the sequencing of pooled libraries was performed on an Illumina MiSeq system using v2 chemistry and 2 × 251 bp reads. The supernatants collected from these cells at 24 hpi were used in subsequent challenge experiments with the respective rocaglates.

### 2.6. Antiviral Activity

To determine and compare the antiviral activities of CR-1-31-B, silvestrol and zotatifin, MRC-5 or Vero E6 cells were inoculated with the respective virus at a MOI of 0.1 at 33 °C (HCoV-229E; SARS-CoV-2) or 37 °C (Middle East respiratory syndrome coronavirus (MERS-CoV)). After 1 h, the inoculum was removed, and cells were incubated with fresh medium containing the inhibitor at increasing concentrations. Supernatants were collected at 24 hpi and virus titers were analyzed by virus plaque assay [19]. To calculate the EC_50_ values, the virus titer determined for virus-infected cells treated with DMSO only was set to 100%, and titers obtained for treated cells were normalized to this value. EC_50_ values were calculated by non-linear regression analysis using GraphPad Prism 6.0 (GraphPad Software). For the infection of primary human airway epithelial cells, the apical surface was washed three times with PBS and cells were infected with MERS-CoV (MOI = 3). The inoculum was removed after 1 h and the medium in the basal compartment was replaced with a medium containing the indicated inhibitor concentrations. At the indicated time points, the apical surface of the cells was incubated with 100 µL/well PBS for 15 min and virus titers in the supernatants were determined by virus plaque assay. Challenge experiments were performed to assess potentially existing differences between non-passaged (p0) and serially passaged (p15) viruses in their viral replication efficiency in cells grown in rocaglate-containing culture medium. To this end, MRC-5 cells were infected with individual p15 and p0 virus isolates, respectively, at an MOI of 0.1 and kept in a medium containing the indicated concentrations of silvestrol, CR-1-31-B or in a medium without these compounds. Viral titers at 24 hpi were determined by plaque assay.

### 2.7. Dual-Luciferase Reporter Assay

The dual-luciferase reporter assay was done as described previously [19,20,21]. For rocaglate treatment, the highest non-cytotoxic concentration (previously determined as 10 nM in a WST-1 assay [19,20,21]) was used. A second concentration of 5 nM was additionally used to substantiate the observed effects. All experiments were performed in at least three independent replicates.

### 2.8. Thermal Shift Assay

Thermal shift assays were performed by incubating 5 µM of recombinant human eIF4AI (19–406) with 50 µM of poly (AG)_5_ RNA, 1 mM AMP-PNP (Roche, Basel, Switzerland), 100 µM of the indicated compound and 75 SYPRO Orange (S6650, Invitrogen, Carlsbad, CA, USA) in the presence of buffer containing 20 mM HEPES-KOH pH 7.5, 300 mM KCl, 5 mM MgCl_2_, 1 mM DTT, 0.1 mM EDTA and 10% glycerol for 10 min at room temperature. Measurements were performed from 10 °C to 95 °C at a 1.6 °C/min ramp rate using the QuantStudio3^TM^ Real-Time PCR system (Applied Biosystems, Waltham, MA, USA) in a MicroAmp^TM^ Fast Optical 96-well plate (0.1 mL, Applied Biosystems, Waltham, MA, USA).

### 2.9. Docking Analysis

Molecular docking was performed using AutoDock, v4.2 [35]. The protein molecules were processed by adding all hydrogen atoms and the merging non-polar hydrogen atoms using AutoDock Tools 1.5.7. Charges were assigned using the Gasteiger method and torsions were fixed for the ligand. The grid box of 60 × 60 × 60 with 3.75 Å was set around the active sites with the x, y and z-dimensions of 46.355, 9.919, 47.473, respectively. The rigid grid box was attained using Autogrid 4, followed by AutoDock with the Lamarckian genetic algorithm to obtain the best docking poses [36]. The docking was performed in two trials, and the average binding energy was calculated. The pose with the best binding affinity was visualized using UCSF Chimera (University of California).

## 3. Results

### 3.1. Serial Passaging of HCoV-229E in the Presence of Rocaglates Does Not Decrease Sensitivity to the Corresponding Rocaglates

**Figure 1:**
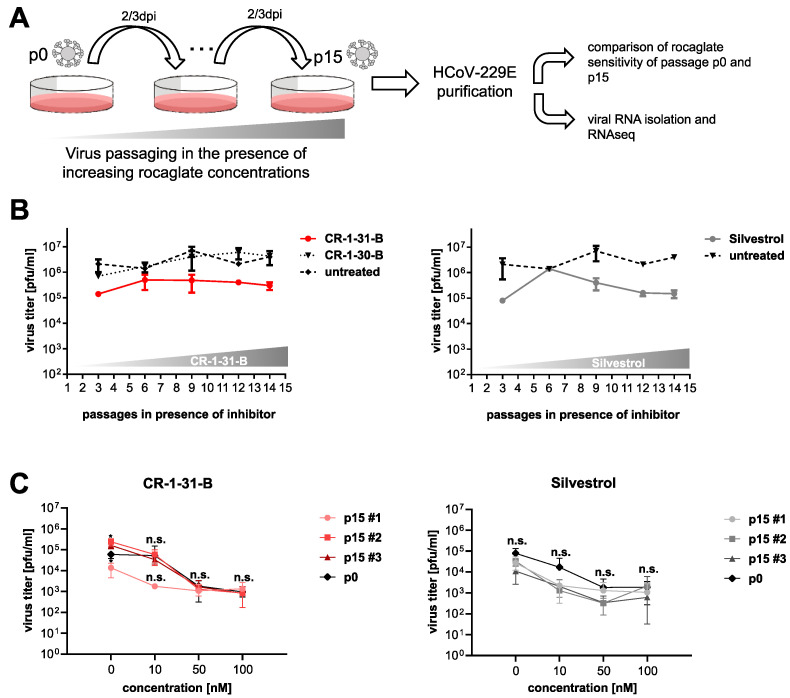
Passaging of HCoV-229E in the presence of increasing concentrations of rocaglates. (**A**) Schematic illustration of workflow. (**B**) MRC-5 cells were infected and treated as shown in (**A**) and titers were determined (*n* = 3). Here, one representative passaging experiment out of three is shown. (**C**) MRC-5 cells were infected with plaque-purified HCoV-229E from p0 (non-passaged) to p15 (serially passaged, obtained as shown in panel (**B**) at an MOI of 0.1 and treated for 24 h either at the indicated concentrations of CR-1-31-B or silvestrol or left untreated for the same time period. Viral titers were then determined using plaque assay (*n* = 3). Significance levels compared to p0 were obtained using the unpaired *t*-test and are indicated as follows: *: *p* < 0.05; n.s.: not significant. Error bars show SD.

To examine the ability of coronaviruses to develop resistance against rocaglates by acquiring mutations in the viral genome, we passaged HCoV-229E in three independent experiments 15 times in the presence of increasing concentrations (from 5 to 10 nM) of rocaglate derivatives, silvestrol or CR-1-31-B (Figure 1A). Corresponding concentrations of the inactive enantiomer CR-1-30-B or a solvent-only sample served as negative controls.

**Figure 2:**
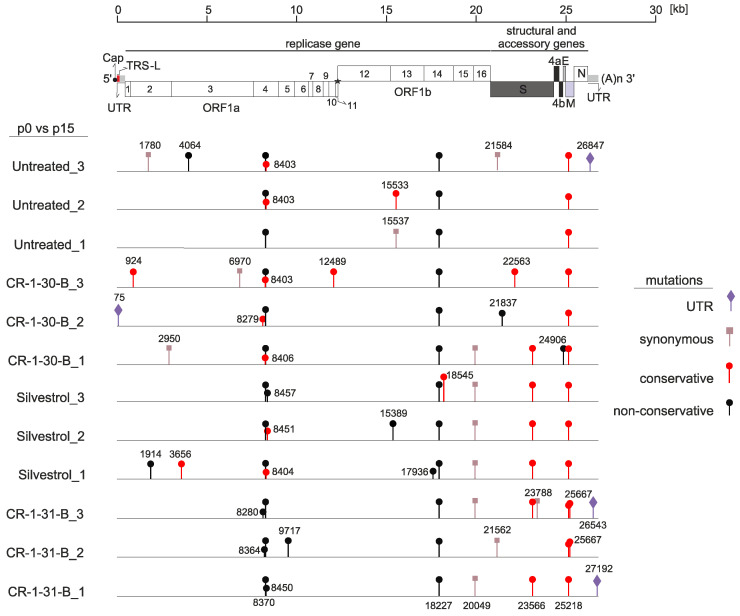
Genetic comparison of input HCoV-229E virus genomes (p0) to obtained p15 virus isolates treated with different rocaglates. CR-1-30-B, the inactive enantiomer of CR-1-31-B, served as a control. MRC-5 cells were infected with the input virus (p0) or the 15 times passaged virus isolates obtained in Figure 1. (MOI = 3). Viral RNA was isolated 24 hpi and sequenced. Single-nucleotide changes are indicated at their location in the virus genome (synonymous: light brown; conservative missense: red; non-conservative missense: black).

As shown exemplarily in Figure 1B, there was no increase of virus replication as a result of serial passaging of the virus in cells propagated in the rocaglate-containing medium. After 15 passages in cell culture, virus-containing supernatants were collected and used to obtain individual isolates by two consecutive end-point dilutions. The individual isolates were subsequently used in a challenge experiment to determine potential differences in drug sensitivity between non-passaged (p0) and serially passaged viruses (p15). As illustrated in Figure 1C, none of the passaged isolates displayed a reduced sensitivity to rocaglate treatment. All p15 isolates replicated with similar efficiency (e.g., CR-1-31-B p15 #2 and #3) or even less efficiently (e.g., CR-1-31-B p15 #1) compared to non-passaged (p0) virus (Figure 1C, black line). Moreover, the genomes of p0 and p15 viruses were sequenced to identify and compare potential mutations that were acquired during in vitro passaging (Figure 2). Most of the mutations were found to be randomly distributed over the virus genome, with very few single-nucleotide polymorphisms (SNPs) in each of the isolates. Besides synonymous mutations (Figure 2, light brown), several non-synonymous mutations (Figure 2, black and red) were identified, including conservative and non-conservative substitutions (by residues with similar or different physico-chemical properties; indicated in red or black, respectively, in Figure 2). Compared to the p0 non-passaged virus, all p15 virus isolates (including the controls) had acquired three non-synonymous mutations in the nsp4, nsp14 and M protein-coding regions, respectively, most likely resulting from a general cell culture adaptation process.

Moreover, one synonymous mutation in nsp15 and one non-synonymous mutation in the spike protein-coding region (position 23566 in the genome), leading to a Glu-to-Asp replacement, could be found in all silvestrol-treated samples and in 2 out of 3 virus isolates passaged in the presence of CR-1-31-B. Considering that (i) this non-synonymous mutation is associated with the connector domain of the spike protein and (ii) generally no reduced drug sensitivity was observed (Figure 1C), it appears unlikely that this represents a rocaglate-specific mutation.

Overall, these findings suggest that HCoV-229E, passaged 15 times in the presence of increasing concentrations of rocaglates, did not acquire mutations that could potentially cause rocaglate resistance in infected cells.

### 3.2. Silvestrol, CR-1-31-B and Zotatifin Show Dose-Dependent Inhibition of In Vitro Translation

**Figure 3:**
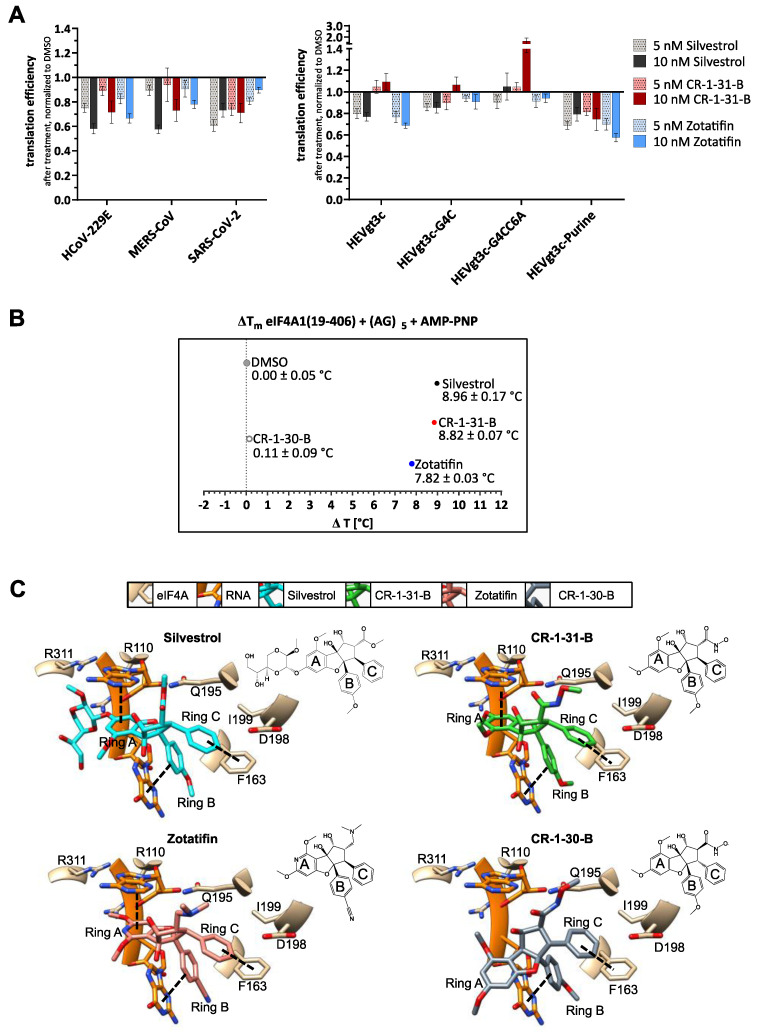
Translation inhibition, thermal shift assay and docking of silvestrol, CR-1-31-B and zotatifin. (**A**) Comparison of the inhibitory effects of silvestrol, CR-1-31-B and zotatifin on reporter gene expression constructs containing 5′-UTRs from coronaviruses HCoV-229E, MERS-CoV and SARS-CoV-2 and analyses of the 5′-UTRs of hepatitis E virus genotype 3c (HEVgt3c) and derivatives thereof with regard to their sensitivity towards silvestrol, CR-1-31-B and zotatifin treatment. Reporter gene expressions were normalized to transfection efficiencies and DMSO controls (*n* ≥ 3). Error bars show SEM. For predicted RNA secondary structures of HEVgt3c 5′-UTR and the respective variants, see Supplementary Figure S1 (**B**) Melting temperature of the eIF4A-polypurine RNA (AG)_5_-AMP-PNP complex in the presence of silvestrol, CR-1-31-B, zotatifin or the inactive enantiomer CR-1-30-B or DMSO to compare the binding affinities of rocaglates to eIF4A-RNA complexes. Numbers indicate the temperature shift compared to DMSO control ± SEM for *n* ≥ 3. (**C**) Predicted binding modes of silvestrol, CR-1-31-B, zotatifin and CR-1-30-B (inactive enantiomer of CR-1-31-B) to the human eIF4A-polypurine RNA (AG)_5_ complex (PDB: 5ZC9) using AutoDock Vina.

Besides resistance analysis, we compared the effects of natural and synthetic rocaglates on in vitro translation efficiency using a dual-luciferase reporter assay [19]. In the presence of the coronavirus 5′-UTRs of HCoV-229E, MERS-CoV and SARS-CoV-2, translation efficiency was sensitive towards silvestrol, CR-1-31-B and zotatifin treatment (Figure 3A). However, the translation inhibition with 10 nM of zotatifin in the presence of the SARS-CoV-2 and the MERS-CoV 5′-UTR appeared to be slightly weaker than with 10 nM of silvestrol or CR-1-31-B. This is not the case for the HCoV-229E reporter construct. The reasons for these differences can so far not be explained and require further investigation.

Since it is known that different rocaglates have different abilities to bind purine-rich sequences [37], we aimed to gain more mechanistic insight into eIF4A inhibition by the rocaglates via investigation of RNA-clamping. For this purpose, we also analyzed the 5′-UTR of HEV (HEVgt3c) that lacks any polypurine stretches but is predicted to form a stable RNA hairpin structure (Figure 3A and Figure S1). Previous studies hypothesized that silvestrol could clamp this HEVgt3c 5′-UTR onto eIF4A with its dioxane moiety, which is excluded from CR-1-31-B that lacks this part of the molecule [20]. Interestingly, we observed translation inhibition of HEVgt3c with zotatifin, suggesting that purine-independent clamping of RNAs onto eIF4A can also be achieved in the absence of a dioxane moiety. By mutating the stem of the HEVgt3c-5′-UTR, we created thermodynamically destabilized variants of HEVgt3c (HEVgt3c-G4C and HEVgt3c-G4CC6A). These weakened RNA structures led to a loss of the inhibitory effects of silvestrol and zotatifin. To further test the relevance of purines, a purine stretch with a length of eight nucleotides was introduced into the 5′-UTR of HEVgt3c (HEVgt3c-Purine, for further details, see Supplementary Figure S1 and [20]). Similar to our previous results, we regain translation inhibition of HEVgt3c-Purine with silvestrol, CR-1-31-B and zotatifin. This demonstrates that with silvestrol and zotatifin either a stable hairpin substrate or a polypurine stretch is sufficient to clamp the RNA onto eIF4A. In contrast, CR-1-31-B shows translation inhibition only in the presence of a polypurine stretch. This is in line with in vitro translation experiments in the presence of CR-1-31-B that indicated the requirement for a stretch of at least 5 purines to elicit inhibition of translation [37].

To further compare the inhibition mechanism, we performed a thermal shift assay to monitor the thermal stability of an eIF4A-(AG)_5_ RNA-rocaglate complex (Figure 3B). The highest thermal shifts were observed in the presence of silvestrol (8.96 ± 0.17 °C) or CR-1-31-B (8.82 ± 0.07 °C). In the presence of zotatifin, the thermal stability of the eIF4A-RNA complex increased by 7.82 ± 0.03 °C, indicating that the complex is somewhat less stable than the complex formed with CR-1-31-B or silvestrol. As expected, the inactive enantiomer CR-1-30-B only showed a thermal shift of 0.11 ± 0.09 °C, confirming that the RNA substrate cannot be efficiently clamped with the (+)-enantiomer.

With regard to the mode of action, we performed docking experiments with silvestrol, zotatifin, CR-1-31-B and CR-1-30-B using the crystal structure of the eIF4A1-AMPPNP-RocA-polypurine RNA complex (PDB: 5ZC9, [38] (Figure 3C)). The three crucial π-π-stacking interactions with the phenylalanine at position 163 of eIF4A, the adenine base of A7, and the guanine base of G8 of the RNA substrate can be formed with all active rocaglates. The inactive enantiomer CR-1-30-B lacks the interaction with A7 of the RNA, explaining the absence of inhibitory activity. In line with the predicted docking poses, only the dioxane moiety of silvestrol can cross the bound RNA substrate and might form additional H-bonds with arginine residues. Therefore, differences in the mode of action of rocaglates might arise from very individual structure-activity relationships (SAR) based on their deviating chemical structures.

### 3.3. Effects on Viability of Silvestrol, CR-1-31-B and Zotatifin in Primary Human Immune Cells

**Figure 4:**
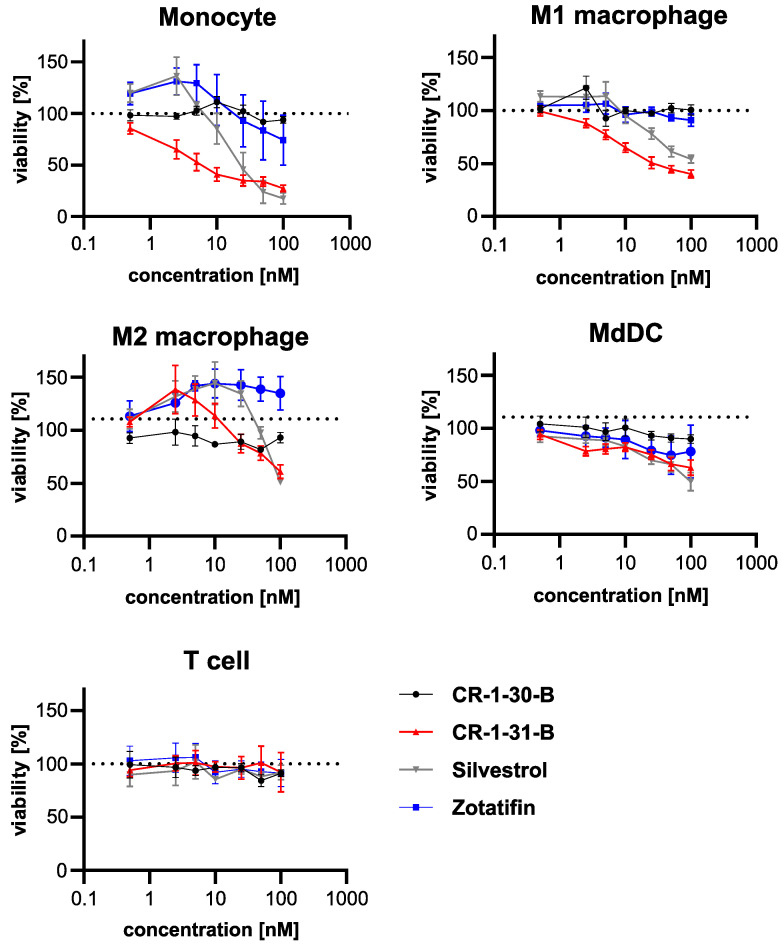
Effects of rocaglates on immune cell viability. Percentage of viable human monocytes, M1/M2 macrophages, MdDCs and T cells in the presence or absence of different concentrations of silvestrol, CR-1-31-B, zotatifin, the inactive enantiomer CR-1-30-B or DMSO control determined by Orangu assay in triplicates. Error bars show SEM with *n* ≥ 3.

Next, the effects of the three rocaglates on the viability of primary human immune cells (monocytes, M1 macrophages, M2 macrophages, monocyte-derived dendritic cells (MdDCs) and T cells) were analyzed (Figure 4). The cytotoxic concentration of silvestrol that reduced the viability of monocytes and M1 macrophages by 50% (CC_50_) was 29 nM and 45.6 nM, respectively. For the treatment with CR-1-31-B, the obtained CC_50_ values for monocytes and M1 macrophages were 2.6 and 8.8 nM, respectively. No significant cytotoxicity could be observed for any of the rocaglates on M2 macrophages, MdDCs and T cells (see Figure 4). However, a concentration of 100 nM silvestrol or 100 nM CR-1-31-B reduced the viability of M2 macrophages and MdDCs by approximately 50%, whereas T cells were not affected (Figure 4, Table 1). Interestingly, 100 nM of zotatifin only reduced the viability of monocytes to approximately 75%. CR-1-30-B, the inactive (+)-enantiomer, had essentially no impact on cell viability at all. These data indicate that the cytotoxic effects of natural and synthetic rocaglates are cell type-dependent and compound-specific.

### 3.4. Zotatifin Reduces Coronavirus Replication In Vitro

**Figure 5:**
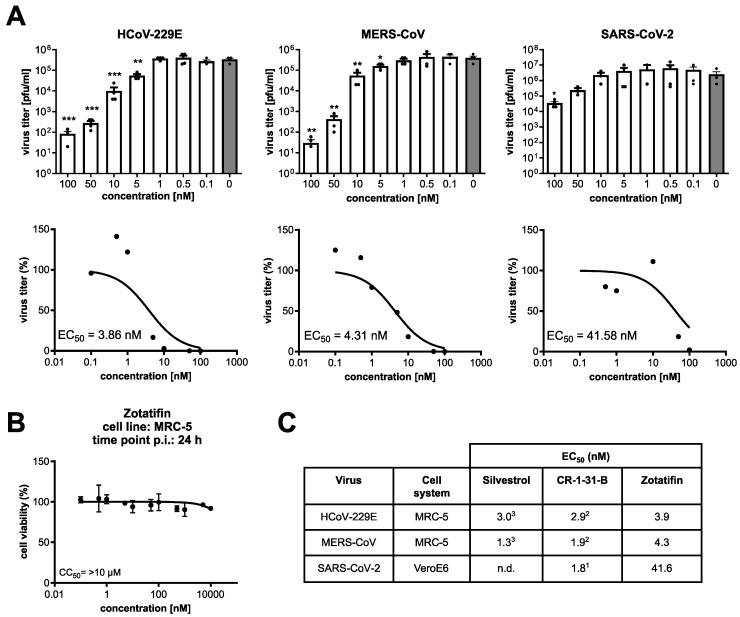
Antiviral activity of zotatifin against different CoVs and comparison with silvestrol and CR-1-31-B. (**A**) MRC-5 or Vero E6 cells were infected with an MOI of 0.1 with HCoV-229E (MRC-5 cells), MERS-CoV (MRC-5 cells) or SARS-CoV-2 (Vero E6 cells) for 24 h in the presence of indicated concentrations of zotatifin or solvent control. Significance levels compared to the results for untreated cells are indicated as follows: *, *p* < 0.05; **, *p* < 0.005; ***, *p* < 0.0005. Error bars show SEM. (*n* ≥ 3). (**B**) Viability assay (MTT assay) of MRC-5 cells treated for 24 h with the indicated concentrations of zotatifin. (**C**) Overview of EC_50_ values against CoVs measured in the present study or reported previously (as described in ^1^ = [21]; ^2^ = [20]; ^3^ = [19]); n.d.: not determined.

We have recently shown that the natural rocaglate silvestrol and the synthetic rocaglate CR-1-31-B efficiently reduce the replication of different coronaviruses in infected cells [19,20,21]. Here, we analyzed the antiviral activity of zotatifin in human fetal lung fibroblasts (MRC-5 cells) infected with HCoV-229E and MERS-CoV, respectively, and Vero E6 cells infected with SARS-CoV-2 (MOI = 0.1). A dose-dependent reduction in virus titers was observed for all three coronaviruses, resulting in EC_50_ values of 3.9 nM for HCoV-229E, 4.3 nM for MERS-CoV and 41.6 nM for SARS-CoV-2 (Figure 5A). Under the conditions used in these experiments, no significant cytotoxicity was observed for up to 10 µM zotatifin (Figure 5B). Taken together, the data demonstrate a similar antiviral potency of zotatifin in comparison to silvestrol and CR-1-31-B (Figure 5C). However, compared to CR-1-31-B, zotatifin was around 20-fold less efficient against SARS-CoV-2, potentially indicating a Vero E6 cell-specific effect (SARS-CoV-2 does not replicate in MRC-5 cells [30]).

### 3.5. Synthetic Rocaglates Zotatifin and CR-1-31-B Efficiently Reduce Mers-Cov Replication in a Human Airway Epithelial Cell Model

**Figure 6:**
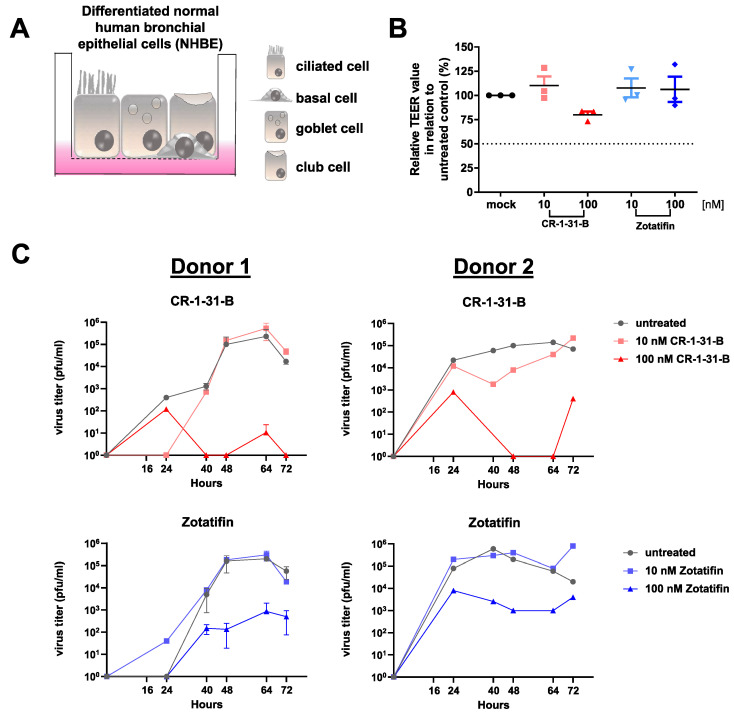
Comparison of antiviral effects of CR-1-31-B and zotatifin using human bronchial epithelial cells infected with MERS-CoV. (**A**) Human bronchial epithelial cells were cultivated and differentiated under the air–liquid interface into different airway epithelial cell types (basal, ciliated, club and goblet cells) and used to assess antiviral effects of the respective compounds. (**B**) Trans-epithelial electrical resistance (TEER) measurement of NHBE cells treated for 72 h with the indicated compounds. (**C**) MERS-CoV titers of infected NHBE cells from two different donors collected at the indicated time points p.i. and treated with CR-1-31-B or zotatifin (each at 10 and 100 nM) in comparison with the solvent control.

To evaluate and compare the antiviral potency of zotatifin in a more relevant setting, we used primary normal human bronchial epithelial (NHBE) cells. These cells, cultured and differentiated under air–liquid interface conditions, serve as a universal model system to study the replication of respiratory viruses, including coronaviruses. Differentiated NHBE cells (Figure 6A), produced from cells obtained from two healthy donors, were infected with MERS-CoV in the presence of the indicated rocaglates or solvent control.

The degree of cell layer integrity as an indication of cytotoxic effects of the used compounds was analyzed 72 h post-treatment by measuring the trans-epithelial electrical resistance (TEER; [39]). Except for 100 nM of CR-1-31-B treatment (~25% signal reduction), no significant cytotoxicity was detectable in the treated NHBE cells compared to the untreated control (Figure 6B). Whereas 10 nM of CR-1-31-B or 10 nM of zotatifin had no significant effect on the replication of MERS-CoV in infected NHBE cells from both donors (Figure 6C), 100 nM of CR-1-31-B reduced viral replication to nearly undetectable levels and 100 nM of zotatifin reduced viral replication by about two orders of magnitude.

## 4. Discussion

Here, we evaluated the ability of coronaviruses to evolve drug-resistant mutations against rocaglates targeting the host factor eIF4A. Interestingly, rocaglate resistance has been reported in cancer cell lines, treated for a prolonged period with rocaglates. As an example, the acute lymphoblastic leukemia cell line 697, treated for 40 weeks with increasing concentrations of silvestrol, obtained drug-resistance by overexpressing the multidrug-resistance protein 1 (MDR1), which is a major cellular efflux transporter [40]. Since in our study only the virus was constantly passaged in the presence of rocaglates, resistances linked to host metabolism were not expected and therefore not followed up.

Viruses passaged 15 times in MRC-5 cells in the presence or absence of increasing concentrations of silvestrol or CR-1-31-B remained sensitive to rocaglates in vitro, thus providing no evidence for the emergence of viral escape mutants in the applied settings upon rocaglate treatment. Among the passaged virus isolates, only a few minor nucleotide changes were identified. One non-synonymous, conservative substitution in the S protein was explicitly enriched in the rocaglate-treated viruses at passage 15. Other mutations identified after p15 of HCoV-229E were not specifically associated with rocaglate treatment, indicating nonspecific selective pressure or cell culture adaptations. The fact that all p15 virus isolates showed no selective advantage in replication over p0 viruses and maintained their sensitivity towards rocaglate treatment, indicates a high barrier for escape mutations against these antiviral compounds targeting the host protein eIF4A. At this stage, we cannot formally exclude that viral adaptations emerge after prolonged passaging.

In comparison with our findings, 13 passages of SARS-CoV-2 in the presence of the DAA remdesivir, which inhibits the viral RNA-dependent-RNA polymerase (RdRp), were reported to lead to partial resistance due to a non-synonymous mutation in the RdRp (E802D) [41]. Similar findings were also reported for other coronaviruses, such as the mouse hepatitis virus (MHV). Here, an increased ability to replicate in the presence of remdesivir was observed after 23 passages. More specifically, two mutations in the RdRp (F476L and V553L) led to a 5.6-fold resistance to remdesivir, based on EC_50_ values. Likewise, homologous substitutions in the genome of SARS-CoV (F480L and V557L) conferred resistance to remdesivir as well [42]. Although these resistance mutations impaired (in the absence of remdesivir) the competitive fitness of the viruses in vitro (SARS-CoV-2 and MHV) and caused attenuation in vivo (SARS-CoV) [41,42], it should be emphasized that DAA-resistant virus mutants can emerge rapidly, which calls for short treatment windows and combination therapies. Against this background, continued efforts to develop HDAs are highly desirable.

We further compared three different rocaglates regarding their potency of translation inhibition in a viral 5′-UTR-sensitive reporter gene expression system. Moreover, we evaluated their capacity to clamp the eIF4A-polypurine (AG)_5_ RNA complex using a thermal shift assay. In line with our results on the antiviral activities of rocaglates, we observed inhibition of translation in the presence of coronavirus 5′-UTRs with all three rocaglates (Figure 3A). Silvestrol, with its additional dioxane moiety, but surprisingly also zotatifin, were able to clamp stable RNA hairpin structures in a polypurine-independent manner, whereas CR-1-31-B only showed inhibition of translation in the presence of a polypurine stretch, which is in line with data obtained in previous studies [20,37] (Figure 3A and Figure S1). Apart from that, reduced RNA hairpin stability led to a loss of the inhibitory effects of all tested rocaglates, indicating that the thermodynamic stability of the RNA substrate is crucial for purine-independent inhibition (Figure 3A and Figure S1). The thermal shift assay results further revealed that eIF4A-polypurine (AG)_5_ complexes are most stable with silvestrol and CR-1-31-B. In the presence of zotatifin, the complexes showed a slightly reduced thermal stability, which might explain the somewhat lower antiviral activity of zotatifin observed here. Our observation of polypurine-independent RNA-clamping, mediated by zotatifin and silvestrol in the reporter assay, might indicate a broader spectrum of viral and also cellular mRNA substrates that are affected by these rocaglates.

Regarding cellular toxicity in human immune cells, all three rocaglates showed no significant cytotoxicity in T cells, dendritic cells and M2 macrophages. Moreover, zotatifin seemed to be better tolerated than CR-1-31-B and silvestrol by monocytes and M1 macrophages. In general, silvestrol is known to influence the inflammatory status of immune cells depending on the cell type and activation status [34]. If this is also the case for zotatifin and CR-1-31-B has not been investigated so far. However, modulating the immune response after viral infection is of outstanding importance for the development of antiviral drugs that can be applied in the later stages of a viral disease. Therefore, rocaglates might be very interesting therapeutic compounds due to their dual effects on virus replication and on modulation of the immune response. 

The antiviral activities of all three tested rocaglates are very similar in MRC-5 cells infected with HCoV-229E or MERS-CoV (Figure 5). However, for Vero E6 cells infected with SARS-CoV-2, the EC_50_ value of zotatifin was about 20-fold higher than previously observed for CR-1-31-B. Further studies are required to determine if these differences are linked to the different cell systems used or result from specific properties of the SARS-CoV-2 isolate (Figure 5C) [43]. It should be noted that a previous study by Gordon et al. reported an EC_90_ for zotatifin of 37 nM against SARS-CoV-2 in Vero E6 cells [44].

To further assess the antiviral potential of the two synthetic rocaglates zotatifin and CR-1-31-B, we analyzed their effects in an infection-relevant ex vivo cell system, namely differentiated human bronchial epithelial cells. This system allows the investigation of inhaled pathogens, including respiratory viruses, on a pseudostratified epithelium, including goblet cells, ciliated cells, club cells and basal cells [45]. Notably, the antiviral effect of CR-1-31-B as well as of zotatifin against MERS-CoV replication could be confirmed in this model system. Interestingly, no major inhibition of MERS-CoV replication was observed for both synthetic rocaglates at a concentration of 10 nM, in contrast to HCoV-229E [20] and SARS-CoV-2 [21]. Our data suggest a lower sensitivity of MERS-CoV to rocaglate treatment in this ex vivo primary cell system. Overall, CR-1-31-B appears to be more effective but also slightly more toxic than zotatifin (Figure 6B,C).

## 5. Conclusions

Our results clearly show that treatment with rocaglates is a potent antiviral approach against different coronaviruses. Our findings support the notion that targeting the host factor eIF4A by rocaglates is associated with a low risk of rapidly emerging virus escape mutants. In addition, we were able to show that rocaglates have differential cytotoxic effects on different human immune cell subpopulations. Application via an aerosol into the respiratory tract of infected organisms (instead of a systemic application) may reduce the uptake of rocaglates into blood cells and thereby reduce the risk of potential toxic side effects. Another advantage of this application route is that the first-pass effect of the liver can be omitted. Therefore, a local application of rocaglates should be the preferred route for future studies that aim at combating respiratory viruses.

## Figures and Tables

**Table 1:** Overview of cytotoxic concentration 50 (CC_50_) of the indicated compounds in corresponding cell systems.

Cell Type	CC_50_ (nM)			
	Silvestrol	CR-1-31-B	CR-1-30-B	Zotatifin
monocytes	29.0	2.6	>100	78.1
M1 macrophages	45.6	8.8	>100	>100
M2 macrophages	>100	>100	>100	>100
dendritic cells	>100	>100	>100	>100
T cells	>100	>100	>100	>100

## Data Availability

All data are available from the corresponding authors upon reasonable request.

## Supplementary Materials

The following supporting information can be downloaded at: https://www.mdpi.com/article/10.3390/v14030519/s1, Figure S1: (A) Effects of 5 nM and 10 nM Silvestrol, CR-1-31-B and Zotatifin on reporter gene expression. The 5′-UTR of the human ß-Globin mRNA and the unstructured (AC)_15_ sequence serve as negative controls, while the (AG)_15_ polypurine sequence servesas a positive control. Reporter gene expressions were normalized to transfection efficiencies and DMSO controls (*n* > 3). (B) Predicted RNA secondary structures of ß-Globin 5′-UTR, (AC)_15_, (AG)_15_ and HEVgt3c 5′-UTR constructs. The secondary structures were predicted using the RNAfold web server (University of Vienna) and structures with minimal free energy are shown.
