# Supplementary material for: Rocaglates as Antivirals: Comparing the Effects on Viral Resistance, Anti-Coronaviral Activity, RNA-Clamping on eIF4A and Immune Cell Toxicity
Source: Viruses. 2022 Mar 3;14(3):519. doi: 10.3390/v14030519 (PMC8950828; doi:10.3390/v14030519)
Supplement: Supplementary file 1 [file viruses-1603177-supplementary.pdf › viruses-1603177-supplementary.pdf]

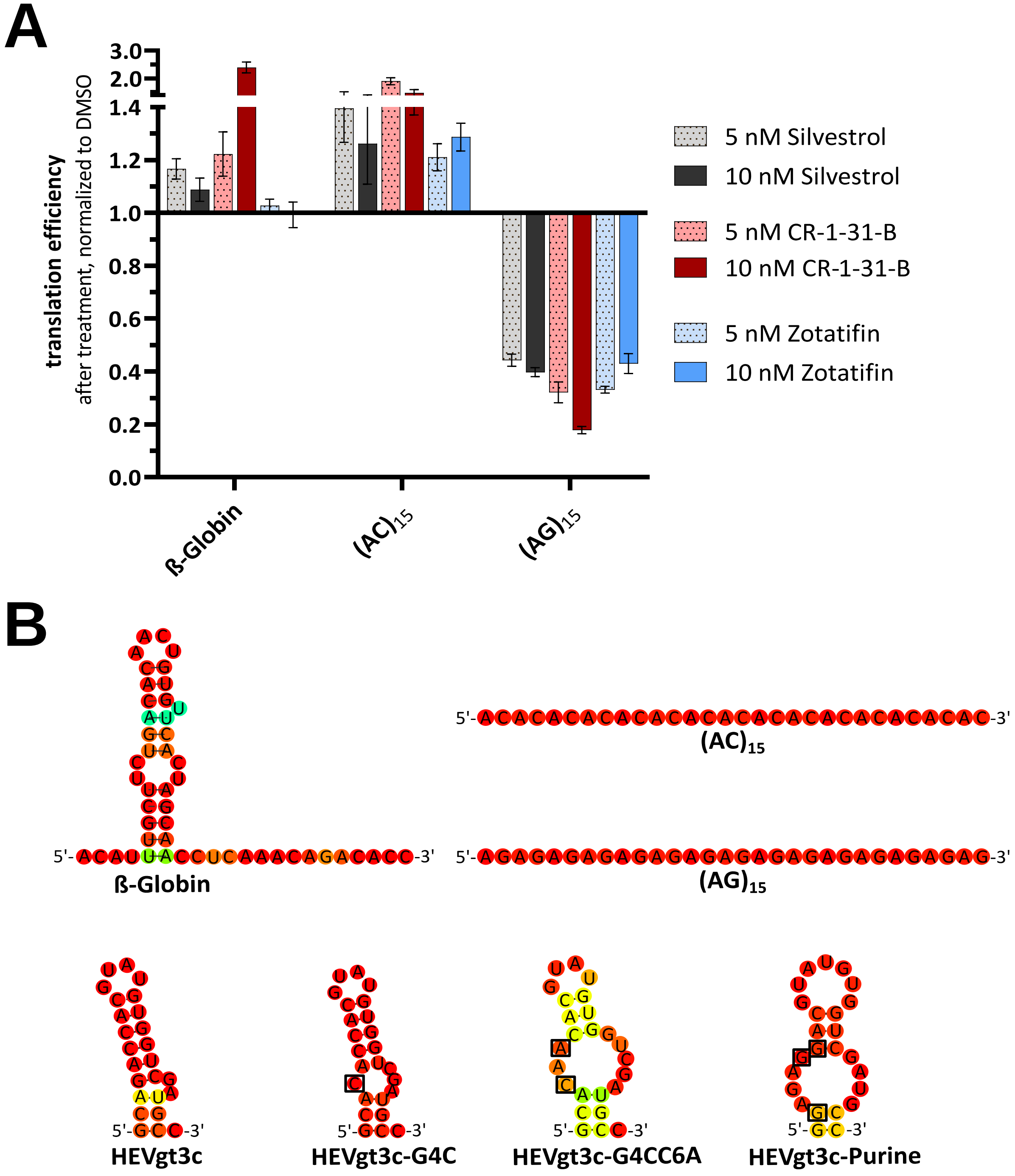

Suppl. Figure 1. (A) Effects of 5 nM and 10 nM Silvestrol, CR-1-31-B and Zotatifin on reporter gene expression. The 5'-UTR of the human ß-Globin mRNA and the unstructured $(AC)_{15}$ sequence serve as negative controls, while the $(AG)_{15}$ polypurine sequence serves as a positive control. Reporter gene expressions were normalized to transfection efficiencies and DMSO controls (n>3). (B) Predicted RNA secondary structures of ß-Globin 5'-UTR, $(AC)_{15}$, $(AG)_{15}$ and HEVgt3c 5'-UTR constructs. The secondary structures were predicted using the RNAfold web server (University of Vienna) and structures with minimal free energy are shown.
